# Landscape Composition and Soil Physical–Chemical Properties Drive the Assemblages of Bacteria and Fungi in Conventional Vegetable Fields

**DOI:** 10.3390/microorganisms10061202

**Published:** 2022-06-12

**Authors:** Uttam Kumar, Hafiz Sohaib Ahmed Saqib, Waqar Islam, Parmar Prashant, Nidhibahen Patel, Wei Chen, Feiying Yang, Minsheng You, Weiyi He

**Affiliations:** 1 State Key Laboratory of Ecological Pest Control for Fujian and Taiwan Crops, Institute of Applied Ecology, Fujian Agriculture and Forestry University, Fuzhou 350002, China; uttam5454@gmail.com (U.K.); prashantparmar093@gmail.com (P.P.); nidhipatel6372@gmail.com (N.P.); blankerchen@foxmail.com (W.C.); 18059141865@163.com (F.Y.); msyou@fafu.edu.cn (M.Y.); 2 Joint International Research Laboratory of Ecological Pest Control, Ministry of Education, Fuzhou 350002, China; 3 Ministerial and Provincial Joint Innovation Centre for Safety Production of Cross-Strait Crops, Fujian Agriculture and Forestry University, Fuzhou 350002, China; 4 Guangdong Provincial Key Laboratory of Marine Biology, College of Science, Shantou University, Shantou 515063, China; 5 Xinjiang Key Laboratory of Desert Plant Roots Ecology and Vegetation Restoration, Xinjiang Institute of Ecology and Geography, Chinese Academy of Sciences, Urumqi 830011, China; ddoapsial@yahoo.com; 6 University of Chinese Academy of Sciences, Beijing 100049, China; 7 Institute of Horticultural Sciences, Jiangxi Academy of Agricultural Sciences, Nanchang 330200, China

**Keywords:** agroecosystem, microbiome, high throughput sequencing, rhizosphere, soil-microbe interactions

## Abstract

The soil microbiome is crucial for improving the services and functioning of agroecosystems. Numerous studies have demonstrated the potential of soil physical–chemical properties in driving the belowground microbial assemblages in different agroecosystems. However, not much is known about the assemblage of bacteria and fungi in response to soil physical–chemical properties and the surrounding landscape composition in different vegetable fields of a highly intensive agricultural system. Here, we investigated the effects of soil physical–chemical properties and landscape composition on the community trends of bacteria and fungi in two different soil compartments (bulk and rhizospheric soils) of two different brassica crop types (Chinese cabbage and flower cabbage). The results revealed that bulk soil had a higher alpha diversity of both bacteria and fungi than rhizospheric soil. Each of the soil physical–chemical properties and landscape compositions contributed differently to driving the community structure of distinct bacterial and fungal taxa in both soil compartments and crop types. The higher proportions of forest, grassland, and cultivated land, along with the higher amount of soil calcium in flower cabbage fields, promote the assemblage of Gammaproteobacteria, Actinobacteria, Oxyophotobacteria, Agaricomycetes, and Eurotiomycetes. On the other hand, in Chinese cabbage fields, the increased amounts of iron, zinc, and manganese in the soil together with higher proportions of non-brassica crops in the surrounding landscape strongly support the assemblage of Deltaproteobacteria, Gemmatimonadetes, Bacilli, Clostridia, Alphaproteobacteria, an unknown bacterial species Subgroup-6, Mortierellomycetes, Rhizophlyctidomycetes, and Chytridiomycetes. The findings of this study provide the most comprehensive, comparative, and novel insights related to the bacterial and fungal responses in a highly intensive vegetable growing system for the improvement of the soil fertility and structure. These are important clues for the identification of key bacteria and fungi contributing to the plant–environment interactions and are of a practical significance for landscape-based ecological pest management.

## 1. Introduction

Crucial environmental services provided by soil microbes include nutrient cycling, carbon storage, and soil remediation [1,2]. Changes in land composition affect the assemblage of soil microorganisms, which has serious ramifications for crop nutrition and health [3,4]. For example, forest cover, grassland, and cultivated land in the landscape composition strongly correlate with soil physical–chemical properties [5,6] and bacterial [7,8] and fungal taxa [9]. Furthermore, root exudates such as amino acids, phenolic compounds, and mucilage have a significant impact on the soil microbial communities [10]. Consequently, the rhizosphere is the home of microorganisms [11,12].

The rhizosphere is a compact zone of soil surrounded by plant roots [13,14], and is a habitat for microorganisms that play a role in complicated biological and ecological interactions [15], as well as one of Earth’s most dynamic interfaces [16]. Plants have the ability to influence the rhizosphere microbial community composition through altering soil pH, increasing nutrition, and reducing competition for beneficial microbes by secreting chemicals that prevent harmful relationships [17]. The rhizospheric microbes, in turn, offer mineral nutrients to plants, help plants endure salt and heat, operate as phytopathogen protectors, boost plant immune systems, and improve plant growth by interacting with phytohormone signaling [18].

Landscape heterogeneity and plant species have been demonstrated to make a substantial difference on rhizosphere microorganisms [19]. The rhizospheric microbial community is primarily drawn from the bulk soil community [20]. As a result, the assembly and eventual composition of the rhizosphere are expected to be influenced by changes in the bulk soil [21]. Understanding the interactions between microorganisms and their host plants in varied landscape compositions would provide researchers a better understanding of the primary elements that can influence microbial prevalence and community structure. Many previous studies have highlighted the impact of change in single landscape composition such as grassland [22], forest [23], and cultivated or arable land [24] on microbial diversity. A combined effect of different landscape composition in the proximity of agricultural lands on soil bacterial and fungal diversity are not well studied. Other than this, there is a growing concern that growing non-mycorrhizal crops and the heavy use of intensive vegetable growing systems will have a negative impact on the microbial diversity [25].

Soil physical–chemical properties also have a major impact on the microbial communities [5,6]. For instance, many of the studies have shown that there was a decrement in the soil microbial diversity with the amount of nitrogen (N) in the soil [26]. Other than this, specific microbial taxa (at phylum and class levels) have shown a strong correlation with many different kinds of soil physical–chemical properties [27,28].

High throughput sequencing has unprecedented advantages to study the composition of soil microbial communities. Therefore, here we employed the Illumina sequencing platform to understand the soil microbial assemblage in brassica vegetation under different landscape gradients. We especially aimed at determining whether the diversity and community compositions of the soil microbiome vary across varying landscape compositions, physical–chemical properties, and different crop types. We hypothesized that (a) landscape composition and crop type will have a significant impact on the bacterial and fungal community composition in soil, and (b) the bacterial and fungal diversity will be higher in the bulk soil than in the rhizospheric soil.

## 2. Materials and Methods

### 2.1. Study Area and Landscape Analysis

**Figure 1:**
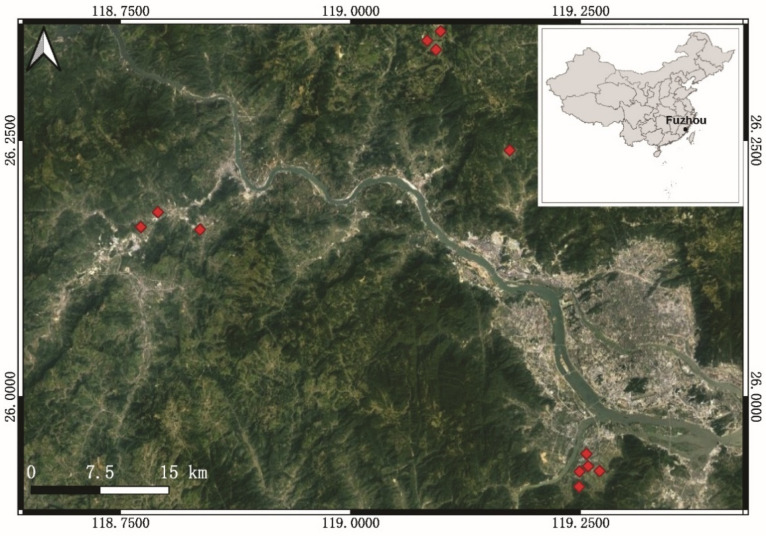
Geographical mapping of sampling sites in the region of Fuzhou city using QGIS. China map (white background in the upper right corner) was drawn using R software.

To represent the range of simple and complex surrounding landscapes, vegetable fields near Fuzhou city in Fujian Province, Southeastern China (N26.00–N26.2500, E118.75–E119.25) were chosen. Each of the 12 fields had a surface area of about 1300–2000 m^2^ and a distance of at least 1 km between them (Figure 1). All sites were conventionally managed in each location, including the use of fertilizers, pesticides (i.e., imidacloprid and Bt), and fungicide (i.e., chlorothalonil). Aerial photos of the landscape distribution in each field were taken by a drone (PHANTOM 4, Shenzhen Dajiang Baiwang Technology Co., Ltd., China) within 500 m of the sampling fields to investigate the landscape distribution. Aerial photos were used to measure the proportions of different vegetation types in a 500-meter- radius (Table S1). We used QGIS 3.4 to map grassland, woodland, urban areas (e.g., residential land, greenhouses, and highways), water surfaces (e.g., rivers, minor steam, and ponds), cruciferous vegetables, and non-cruciferous vegetables (e.g., pepper, eggplant, corn, and bean). By splitting the 500-m-radius landscape surrounding the focal region into five 100-m-distance circular buffer circles, the proportions of different vegetation types were calculated at multiple spatial scales (Figure S1).

### 2.2. Soil Sample Collection and Characterization

Samples of soil were taken on 16–17 November 2019 from 12 different sites near the Fuzhou city based on landscape gradient composition and brassica crop type (Chinese cabbage (CC) and flower cabbage (FC)), including three replicates at each site for bulk and rhizospheric soils (Figure S2, Table S2). A total of 72 soil samples were obtained, including bulk and rhizospheric soils. All soil samples were sieved (2 mm) immediately to eliminate visible roots, plant remains, and stones. To assess soil chemical characteristics, the remaining soil was air-dried and crushed, while a part of each soil sample was placed in a 50 mL centrifuge tube and stored at −80 °C to evaluate the microbial communities. CC and FC bulk soil samples are referred to as CC-BuS and FC-BuS, respectively, whereas CC and FC rhizospheric soil samples are referred to as CC-RiS and FC-RiS, respectively.

From each field site, three CC and FC plants were chosen at random. The bulk soil samples were taken from the same field where the plants were not grown, and the distance from the plants was variable from 50 to 100 cm. Three separate soil cores were obtained at a depth of 0–20 cm for each bulk soil sample with two from both ends and one from the plot’s center. For rhizospheric soil, samples were taken from a 10 cm fragment of three randomly chosen root systems, which corresponds to a soil depth of 5–15 cm, using ethanol-sterilized scissors. The root fragments were chopped into 3 cm pieces and washed four times with sterile ddH_2_O in 50 mL Falcon tubes by shaking vigorously. To characterize the rhizospheric soil samples, the wash fractions were centrifuged for 5 min at 3220× *g*. The supernatant was discarded, and the precipitates were air-dried and stored at −80 °C until further use.

### 2.3. Determination of Soil Chemical Elements

The alkaline hydrolysis–diffusion method was used to determine the amount of nitrogen (N) available in the soil [29]. A sodium bicarbonate extraction-molybdenum-antimony anti-colorimetric test was used to evaluate the amount of phosphorus (P) [29]. The ammonium acetate exchange–flame photometric method was used to determine the amount of potassium (K) [30]. Soil exchangeable calcium (Ca) and magnesium (Mg) was analyzed by ammonium acetate exchange–atomic absorption spectrophotometer [31]. A DTPA extraction–atomic absorption spectrophotometer was used to quantify the amount of iron (Fe), manganese (Mn), copper (Cu), and zinc (Zn) [32]. Variation inflation factors (VIFs) for Cu and Mg was greater than 10, which shows collinearity with other factors. As a result, these were removed from the final analysis (Table S2 and Table 1).

### 2.4. Soil DNA Extraction, High-Throughput Sequencing, and Data Analysis

The fungal and bacterial communities were compared using the Illumina HiSeq 2500 high-throughput sequencing method (BioMarker Technologies Corporation, Beijing, China). The Power Soil DNA Isolation Kit was used to recover soil DNA from frozen soil samples according to the manufacturer’s instructions (MoBio Laboratories, Carlsbad, CA, USA). The fungal ITS1 barcode region was amplified using the ITS1 forward primer (5′-CTTGGTCATTTAGGAAGTAA-3′) along with the ITS2 reverse primer (5′-GCTGCGTTCTTCATCGATGC-3′), while the bacterial V3–V4 barcode region was amplified with the “338” forward primer (5′-ACTCCTACGGGAGGCAGCA-3′) and the “806” reverse primer (5′-GGACTACHVGGGTWTCTAAT-3′). The PCR conditions were as follows: denaturation at 95 °C for 5 min; 25 cycles of 95 °C for 30 s, 50 °C for 30 s, and 72 °C for 40 s; and final extension at 72 °C for 7 min. A GeneJET Gel Extraction Kit (Thermo Scientific, Waltham, MA, USA) was utilized to purify the target amplicon, which was then quantified using a Qubit dsDNA HS Assay Kit (Life Technologies, Carlsbad, CA, USA). The raw paired-end reads were merged using FLASH (v.1.2.11) [33], and QIIME (v.1.8.0) [34] was used to analyze them. To analyze the microbial diversity information of the samples, clean tags were grouped at a 97 percent sequence alignment level using USEARCH in QIIME [35]. Different operational taxonomic units (OTUs) were obtained [36], which were then classified and annotated using the SILVA (bacteria) and UNITE (fungi) taxonomy databases. Rarefaction curves were computed and plotted using R (v.3.8.2). Sequencing data were deposited in the NCBI (https://www.ncbi.nlm.nih.gov/, accessed on 18 September 2021) database under accession codes SAMN21466482-SAMN21466517 and SAMN21467089-SAMN21467124 for 16S and SAMN21467213-SAMN21467248 and SAMN21467258-SAMN21467293 for ITS.

### 2.5. Statistical Analysis

The linear response of soil physical–chemical properties for both soil fractions and crop types were studied using a linear regression model. After that, analysis of variance (ANOVA) and Fisher’s least significant difference (LSD) post-hoc test were done to perform the pair-wise comparisons [37]. Phyloseq [38] and microbiome seq [39] programs in R were used in order to determine the relative abundance of the top 15 microbial community types across all samples. The linear response of each taxon for soil percentage and crop type was then examined. In each sample, the ACE, Chao1 [40], and Shannon–Wiener [41] diversity indices were measured, and their linear responses for both soil fraction and crop type were tested using linear regression models, which were then followed by ANOVA and the LSD post-hoc tests. The microbial beta-diversity was determined based on the identified OTUs using non-metric multidimensional scaling (NMDS) and the Bray–Curtis distance matrix between the samples. Furthermore, using the Adonis and analysis of similarity (ANOSIM) tests in the R package vegan, permutational multivariate analysis of variance (PERMANOVA) was used to analyze the influence of soil percentage and crop type on the Bray–Curtis dissimilarity matrix after 999 permutations [42].

A multivariate technique using a redundancy analysis (RDA) was used to reveal each taxa’s linear response to numerous landscape features. Community metrics were Hellinger converted prior to RDA. Thanks to this change, we can now apply ordination methods to datasets with a lot of zeros. VIFs were used to measure the goodness of fit of each RDA model. Environmental variables with VIFs > 10 were collinear with other environmental variables [43]. As a result, they were left out of the final model because they did not significantly explain the variance. An ANOVA-like permutation test was also used to examine the significance of RDA models and environmental factors [44]. R (v.3.6.3) was used to perform all calculations and create visualizations, based on the “phyloseq” [37], “microbiomeSeq” [37], “ggplot2” [45], “vegan” [41], “dplyr”, “forcats”, “multcompView” and “extrafont” packages [46].

## 3. Results

### 3.1. Soil Microbial Community Compositions

At a 97 percent sequence alignment cutoff rate, Illumina Hiseq2500 sequencing generated a total of 4,863,234 (bacteria-16S:V3+V4) and 3,872,450 (fungi-ITS) clean paired reads, classified into 716 bacterial OTUs and 244 fungal OTUs, respectively. From our sequencing data, a total of 30 phyla, 73 classes, 171 orders, 305 families, and 628 genera were used to classify bacterial OTUs. Fungal OTUs were divided into seven phyla, 19 classes, 52 orders, 82 families, and 144 genera in the same way.

**Figure 2:**
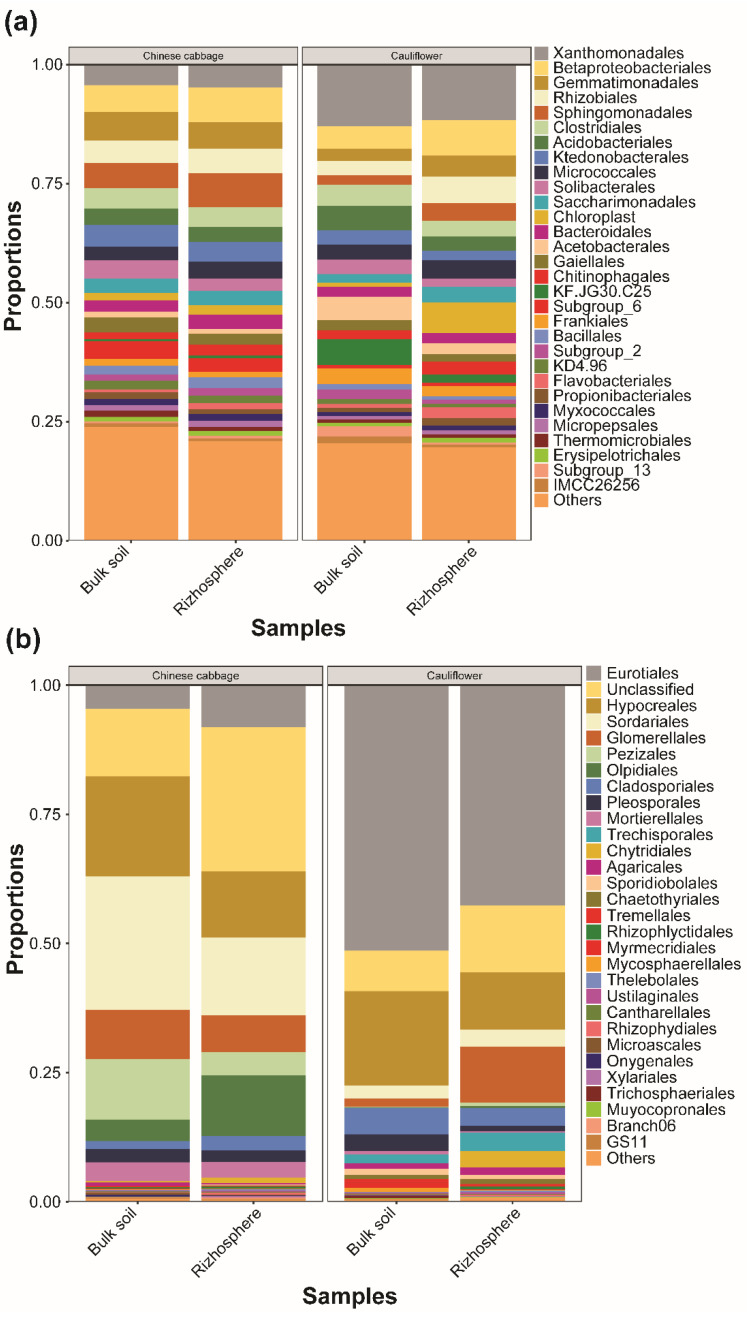
Relative abundance (%) of different bacterial and fungal communities in bulk and rhizospheric soils collected from Chinese cabbage and flower cabbage fields. (**a**) Relative abundance of bacteria at order level and (**b**) relative abundance of fungi at order level.

In the CC field, both the bulk soil and the rhizospheric soil were dominated by Gammaproteobacteria and Alphaproteobacteria classes of bacteria and, respectively, followed by Actinobacteria and Acidobacteriia. Similarly, in the FC fields, Gammaproteobacteria were comparatively higher in proportion than the other classes of bacteria, and the remaining classes of bacteria in the FC fields followed the same pattern as that of CC (Figure S3a). The order Xanthomonadales, Betaproteobacteriales, and Gemmatimonadales were most prominent in both types of the vegetable fields with only one exception of Sphingomonadales, which was present significantly in the rhizospheric soils of CC fields (Figure 2a). In the CC fields, both bulk soil and rhizospheric soil were dominated by Sphingomonadaceae and Gemmatimonadaceae families of bacteria and, respectively, followed by Rhodanobacteraceae and Burkholderiaceae. Similarly, in the FC fields, Rhodanobacteraceae was comparatively higher in proportion for both the soil compartments than the other families of bacteria, and the remaining families of bacteria in the FC fields followed no specific pattern (Figure S3c). The genera *Sphingomonas* and *Chujaibacter* were most prominent in both types of the vegetable fields with only one exception of unclassified KF.JG30.C25, which was present significantly in the rhizospheric soils of FC fields (Figure S3d).

For fungi, Sordriomycetes dominated the CC field in both bulk and rhizospheric soil, while the FC fields were dominated by Eurotiomycetes in both bulk and rhizospheric soils. Other than these, one fourth of the proportion of the fungal community remained unclassified in the rhizospheric soil of CC fields (Figure S3b). The order Eurotiales was the most prominent in both the soils of FC fields followed by Hypocerales and Glomerellales, while in the CC fields, Sodariales was the most prominent order, followed by the Hypocerales and Glomerellales (Figure 2b). For fungi, the family Chaetomiaceae dominated the CC fields in both bulk and rhizospheric soils, while the FC fields were dominated by Aspergillaceae in both bulk and rhizospheric soils. Other than these, one fourth of the fungal family communities remained unclassified in the rhizospheric soil of the CC fields (Figure S3e). The genus *Penicillium* was the most prominent in both the soils of the FC fields followed by *Trichoderma* and *Fusarium*, while in the CC fields, the genus *Fusarium* was the most prominent followed by the *Humicola* and *Olpidium* (Figure S3f).

### 3.2. Variability of Soil Microbial Diversity in Different Local Field Factors

**Figure 3:**
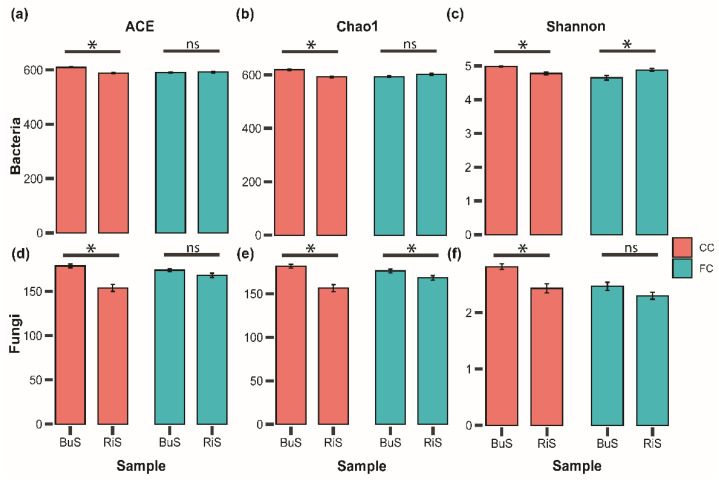
Alpha diversity indices of soil bacteria and fungi. The bar graph depicts alpha diversity (ACE, Chao1, and Shannon indices) of soil bacteria (**a**–**c**) and fungi (**d**–**f**). Error bars with “*” represent significant difference and “ns” represents non-significant differences between different bulk “BuS” and rhizospheric “RiS” soils of Chinese cabbage “CC” and flower cabbage “FC”.

In CC fields, the alpha diversity levels of soil bacteria and fungi were greater in the bulk soil than the rhizospheric soil (Figure 3, Table S3), whereas in the FC fields the chao1 index showed a higher diversity for fungal communities in the bulk soil than the rhizospheric soil (Figure 3e). The Shannon index in the FC fields showed a higher diversity for bacterial communities in the rhizospheric soils than in the bulk soil (Figure 3c).

**Figure 4:**
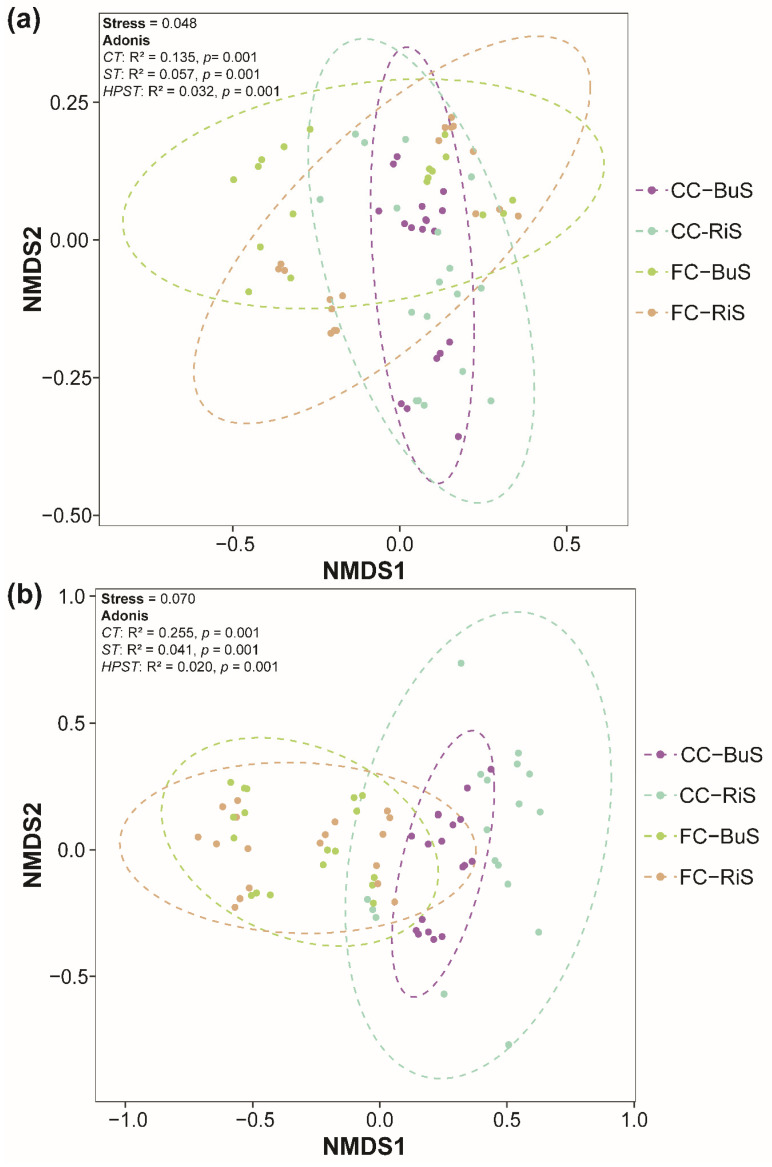
NMDS plots indicating the beta-diversity for (**a**) soil bacteria and (**b**) fungi. Different crop types along with the soil fraction have been designated with different colors. “CC−BuS”: Chinese cabbage and bulk soil, “CC−RiS”: Chinese cabbage and rhizospheric soil, “FC−BuS”: flower cabbage and bulk soil, “FC−RiS”: flower cabbage and rhizospheric soil. Stress values, *p*-values and ANOSIM and Adonis test confirmatory values are indicated on the top of each figure.

According to NMDS analysis, the soil samples from CC and FC fields formed unique groups in the ordination space, with significant differences detected at taxonomic levels using the ADONIS test (Figure 4). Furthermore, the variations in fungal communities between the CC and FC field soils were more pronounced, which indicates that soil fungal communities were more sensitive to the type of brassica crop in the field (Figure 4b). Furthermore, we discovered a considerable fungal community variation between bulk and rhizospheric soils. These differences were greater in fungal communities than in bacterial communities, implying that fungal communities were even more influenced by plant roots (Figure 4a,b).

### 3.3. Integrative Analysis of the Factors Shaping the Soil Microbial Community

**Figure 5:**
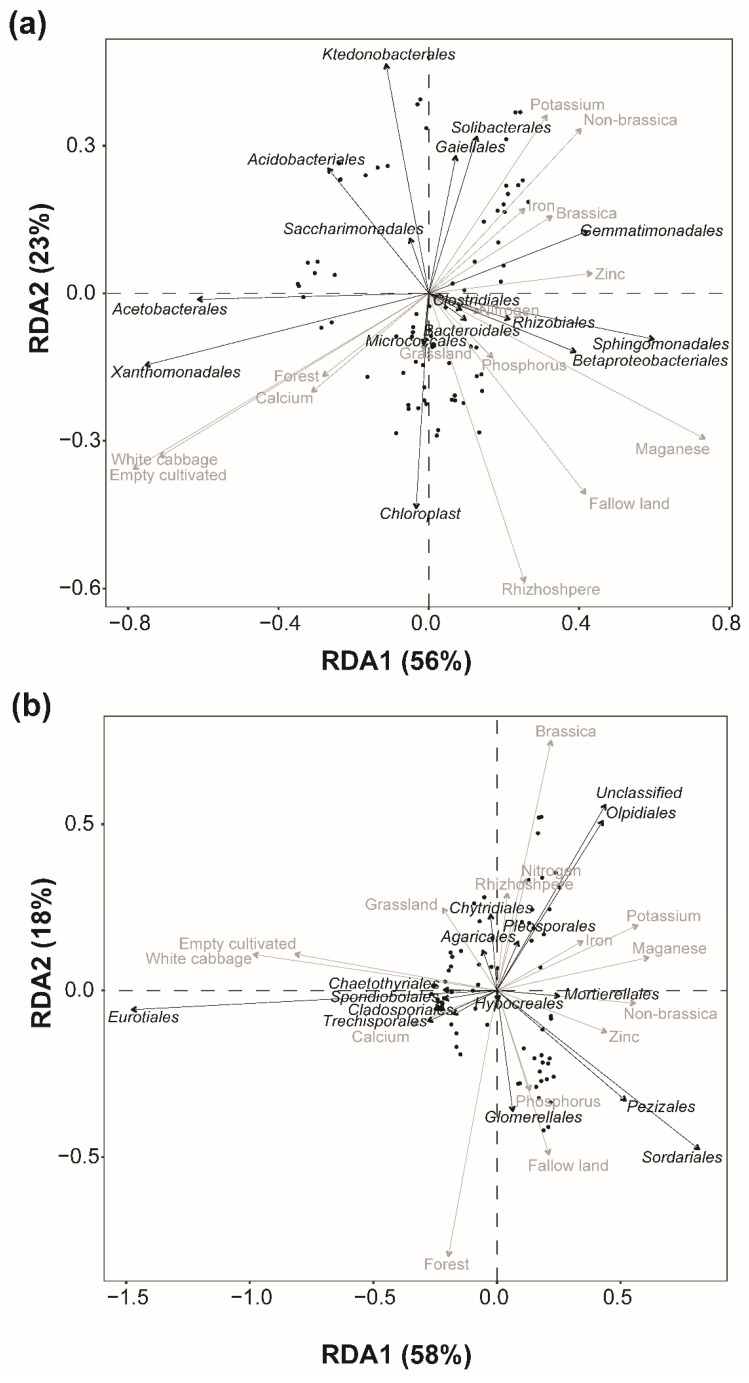
Redundancy analysis (RDA), illustrating the effects of landscape factors, soil physical–chemical properties, soil fraction, and crop type (arrows) on (**a**) soil bacterial orders and (**b**) soil fungal orders. The length and orientation of the arrows show the amount of variance that the explanatory and response variables can explain. The correlations between soil bacterial and fungal classes and explanatory variables are represented by the perpendicular distance between them (<90° = positive correlation, >90° = negative correlation). The strong association is represented by a smaller perpendicular distance.

Redundancy analysis (RDA) was further used to determine the response of microbial structures to the landscape composition and physical–chemical properties. The results showed that landscape composition and physical–chemical properties accounted for 72% and 84% of the total variability in the assemblage of the bacterial and fungal class communities, respectively (Figure S4a,b, Table S4). The results also showed that landscape composition, crop type, soil fraction, and physical–chemical properties together accounted for 79% and 76% of the total variability in the assemblage of the bacterial and fungal order communities, respectively (Figure 5a,b, Table S4). At the family level, landscape composition and physical–chemical properties accounted for 78% and 77% of the total variability in the assemblage of the bacterial and fungal communities, respectively (Figure S4c,d). At the genus level, landscape composition, crop type, soil fraction, and physical–chemical properties together accounted for 82% and 78% of the total variability in the assemblage of the bacterial and fungal communities, respectively (Figure S4e,f). The assemblage of microbial classes, orders, families, and genera was variable and differentially coordinated with crop type, soil fraction, landscape factors, and soil physical–chemical properties.

#### 3.3.1. Effect of Different Factors on Shaping the Soil Bacterial Community

For bacterial classes, Ca, along with the cultivated lands and the grassland portion of the landscape and FC crop type, was predicted to have a significant positive influence on the assemblage of Gammaproteobacteria and Actinobacteria. But K, Fe, and the non-brassica portion of the landscape had a strong negative influence on the assemblage of these classes of bacteria. Similarly, Mn and P, along with the fallow land proportion of the landscape, had a negative influence on the assemblage of Acidobacteria and a positive and strong influence on the assemblage of Alphaproteobacteria and Bacteroidia (Figure S4a, Table 1). In addition, the brassica proportion of the landscape had a positive influence in the assemblage of the class Bacilli, while the fallow land portion of the landscape had a negative influence on the assemblage of the class Acidiomicrobiia (Figure S4a).

For bacterial orders, Ca, along with the cultivated lands and the forest portion of the landscape and FC crop type, was predicted to have a significant positive influence on the assemblage of Xanthomonadales and Acetobacterales. But K, Fe, and the non-brassica portion of the landscape had a strong negative influence on the assemblage of these classes of bacteria. Similarly, Mn and P, along with the fallow land proportion of the landscape and the rhizosphere soil fraction, had a negative influence on the assemblage of Acidobacterales and Ktenobacteriales. While there was a positive and strong influence on the assemblage of Betaproteobacteriales and Sphingomonadales (Figure 5a, Table 1).

For bacterial families, Ca, along with the cultivated lands and the forest portion of the landscape and FC crop type, was predicted to have a significant positive influence on the assemblage of Chitinophagaceae and KF.JG30.C25, while having a negative influence on Gemmatimonadaceae. Similarly, Mn and P, along with the fallow land proportion of the landscape and the rhizosphere soil fraction, had a positive influence on the assemblage of Sphingomonadaceae and Burkholderiaceae (Figure S4c).

For bacterial genera, Ca, along with the cultivated lands and the forest portion of landscape, was predicted to have a significant positive influence on the assemblage of *Chujaibacter* and unclassified KF.JG30.C25, while having a negative influence on *Gemmatimonas* and *Bryobacter*. Similarly, Mn and K, along with the fallow land proportion of the landscape and the rhizospheric soil fraction, had a positive influence on the assemblage of *Sphingomonas* and a negative influence on assemblage of *Rhodanobacter* (Figure S4e).

#### 3.3.2. Effect of Different Factors on Shaping the Soil Fungal Community

For fungal classes, the overall RDA results indicated that crop type (CC versus FC) and soil fractions (bulk versus rhizosphere) were clustered on different ordination axes (Figure S4b). However, the landscape scale factors affected the assemblage differentially. The ordination showed a positive response of Sordariomycetes and Pezizomycetes in terms of assemblage with P, Zn, and the fallow land proportion of the landscape (Figure S4b). Similarly, Chytridiomycetes and Olpidiomycetes also showed a positive response with N, rhizosphere, and brassica proportion in the landscape and a negative response with Ca and forested land, while Agricomycetes and Dothideomycetes showed a positive response with FC crop type, cultivated land, and grassland portion in the landscape (Figure S4b, Table 1).

For fungal orders, the ordination showed a positive response of Sordariales, Glomerellales, and Pezizales in terms of the assemblage with P, Zn, and the fallow land proportion of the landscape (Figure 5b). Similarly, Pleosporales and Olpidiales also showed a positive response with N, rhizosphere, and brassica proportion in the landscape and a negative response with Ca and forested land, while Agaricales showed a positive response with FC crop type, cultivated land, and grassland portion in the landscape (Figure 5b).

For fungal families, the ordination showed a positive response of Chaetomiaceae, Pyronemataceae, and Plectosphaerellaceae in terms of assemblage with P, Zn, and the fallow land proportion of the landscape (Figure S4d). Similarly, Stachybotryaceae and Olpidiaceae also showed a positive response with N, rhizosphere, and brassica proportion in the landscape and a negative response with Ca and forested land, while Chytridiaceae shown a positive response with FC crop type, cultivated land, and grassland portion in the landscape (Figure S4d).

For fungal genera, the ordination showed a positive response of *Colletotrichum*, *Trichocladium,* and *Plectosphaerella* in terms of the assemblage with the P, Zn, and the fallow land proportion of the landscape (Figure S4f). Similarly, *Olpidium* also showed a positive response with the N, rhizosphere, and brassica proportion in the landscape and a negative response with Ca and forested land, while *Trichoderma* and *Penicillium* showed a positive response with FC crop type, cultivated land, and grassland portion in the landscape (Figure S4f).

## 4. Discussion

Our present research contributes to a better understanding of how landscape composition and soil fertility affect soil microbial assemblages. The response of bacterial and fungal populations in two soil components was studied. Like is reported in the previous studies [9,47], the response of the soil microbial assemblage to the landscape composition differs from one community to another. The fungal phyla of Ascomycota (order Eurotiales, Hypocerales, and Glomerellales) and Basidiomycota (order Sodariales) were significantly higher in terms of relative abundance in both the bulk and rhizospheric soils. Ascomycota is the most common and diversified phylum of eukaryotes, as well as the organic substrate decomposers [48,49], and discovered to be the most common fungal phylum in agriculturally cultivated soils [50]. Basidiomycota (order Sodariales and family Chaetomiaceae), being an important decomposer, releases enzymes (peroxide) for degrading plant substances such as cellulose and lignin [51] and increases the overall carbon pool of the soil. The family Chaetomiaceae is known for its cellulolytic members [52].

The bacterial class proteobacteria (order Betaproteobacteriales) was significantly higher in terms of abundance in bulk and rhizospheric soils of both brassica species. This is consistent with previous studies in canola (*Brassica napus*) [53,54], as well as the widely held belief that proteobacteria dominate soil populations [55]. Other than this, members of this class are also involved in solubilizing P, which is valuable in plant growth [56,57]. In brassica species, glucosinolates are one reason for the abundance of proteobacteria [58,59], because glucosinolates generated from roots play a crucial role in determining microbial diversity [60]. Additionally, species from this class are regarded as primary functional microorganisms participating in litter decomposition and transformation. Similarly, Alphaproteobacteria was the most abundant class, with the majority of the members involved in nitrogen fixation [61]. Other than this, Gammaproteobacteria (order Xanthomonadales and Gemmatimonadales) was the second most abundant class, where the majority of species are associated with the phosphorus cycle [62]. This was made evident by the significant and positive correlation between P and the class Gammaproteobacteria. The order Sphingomonadales (genus *Sphingomonas*) was exceptionally higher in the rhizosphere of CC fields and it is widely regarded as best environmentally-friendly approach for P mobilization to plants [63].

The most frequent family detected in our field samples was Gemmatimonadaceae (genus Gemmatimonas), which made up an average of 4% of the soil bacterial communities, corroborating the earlier estimate that Gemmatimonadaceae is one of the most common families found in soil [64]. The prevalence of Gemmatimonadaceae was previously found to respond positively to nitrogen supply [65], which was not observed in our study. Because nitrogen fertiliser is frequent in croplands, the impact of nitrogen is likely to be saturated. In fact, Gemmatimonadaceae responded favourably to potassium in our investigation (Figure S4a). Sphingomonadaceae (genus Sphingomonas) was one of the most abundant bacterial families identified, with an average relative abundance of 2.39% in our study, and its relative abundance was influenced by nitrogen in the soil because the majority of the species belonging to this family are nitrogen fixers in the soil [66]. The abundance of Sphingomonadaceae may also be linked to the utilization of small organic substances resulting from the degradation of humic substances [67]. Organic matter content is highly abundant in the croplands of our study system, and its influence on the abundance of Sphingomonadaceae may have been saturated.

Bacterial and fungal richness (such as observed species), ACE, and the Chao of bulk soil, was substantially higher than that in the rhizospheric soil for both brassica species. Firstly, in response to changes in the landscape compositions and soil environments, the brassica species may release a large amount of root exudates [68], which may be the reason for facilitating the slow reproduction of some microorganisms, resulting in a lower species richness in the rhizospheric soil than in the bulk soil. Secondly, it is likely that the increased nutrient availability or a copiotrophic environment in the rhizosphere may lead to a decrease in species richness by suppressing the oligotrophic fungal groups [48]. Additionally, fungal diversity in the bulk soil was substantially higher than that in the rhizospheric soil, which indicated that the microbial community of the plant rhizosphere, particularly soil fungi, is a subset of the bulk soil ecosystem [20]. Because the bacterial and fungal diversity decreased as the root proximity increased, less diversity in rhizospheric soil compared to bulk soil was not surprising [69].

The soil fraction had a major effect on the bacterial and fungal community structure. First, significant variations in the soil bacterial and fungal population between the bulk and rhizospheric soils were discovered using NMDS analysis, which could be attributed to direct effects by plant root exudates [70], indicating that the bacterial and fungal community assembly is seriously affected by plants. The structure of fungal colonies is strongly influenced by soil chemistry [48]. Combined with RDA results, we can assume that under different landscape compositions, the bacterial and fungal community structure of bulk and rhizospheric soils have undergone significant changes, which can be implicated to the differences in soil chemical properties [71].

Forest and cultivated land in the landscape composition were the two factors that mainly affected the bacterial communities in the bulk soil. For instance, the abundance of the bacterial class Gemmatimonadetes was negatively associated with the forest and cultivated land, which might be due to the higher moisture content of the bulk soil maintained by these two landscape factors [72]. This is consistent with the previous studies, where Gemmatimonadetes were inversely correlated with the moisture content of the soil [64]. It is a big challenge to establish the reason behind the positive correlation of Gemmatimonadetes to the proportion of brassica in the landscape composition because the possible functions of this phylum are difficult to characterize. However, keeping in mind the relative abundance of this phylum in soil, its ecology and functions in the ecosystem must be revealed. Members of the class Bacilli are omnipresent in the agricultural soils and many of the members have a close association with the brassica plants [73,74], which may well explain that brassica had a positive influence on the abundance of Bacilli. The proportion of forest cover near the agricultural fields led to the formation of an anaerobic environment in the bulk soil [75], which in turn decreases the abundance of Deltaproteobacteria. This might be the reason for the negative association of the proportion of forest cover with the abundance of Deltaproteobacteria. A greater availability of soil organic matter due to the proportion of forest cover in the landscape composition could have been the reason for the abundance of Actinobacteria [76]. The members of this class are mainly associated with the decomposition of organic matter such as chitin and cellulose in the soil. The defoliation of the forest plant in the near area of the field might lead to a positive association with the members of the class Actinobacteria [77,78]. Members of the class Ktedonobacteria are involved in degrading the carbohydrates and other polymeric organic compounds into simpler substrates to support the plant growth [79,80]. The proportion of fallow land in the landscape composition is free from the broadleaved forests, members of class Acidimicrobiia are generally involved in the decomposition of broadleaved forests. This might be the reason for the negative correlation of fallow land with the abundance of the class Acidimicrobiia.

In our study, P was positively correlated with the class Pezizomycetes (phylum Ascomycota). In a previous study, the researchers discovered a similar correlation between P and Pezizomycetes [81] and a negative association of P with unclassified communities of the phylum Basidiomycota [47,50], where members of the phylum Ascomycota were abundant and Basidiomycota were few in the soils with a high and low concentration of P, respectively. These results also suggest that an important role is played by the fungal communities in the utilization and absorption of soil P by the plants. Similarly, Ca is also an important element in the regulation of fungal cells, especially in the phylum Basidiomycota [82]. Soil K had a negative correlation with members of the phylum Basidiomycota, which is against the findings of the previous research where K and members of the phylum Basidiomycota showed a positive correlation [83]. Soil Zn is one of the essential metals for fungal growth, differentiation, and metabolism [84]. Although our findings show that landscape composition, crop type, and soil physical–chemical properties were the primary determinants of soil bacterial and fungal diversity, the impact of previous crops, human disturbance, and fertilizer application must all be considered and can be further investigated. In the brassica agroecosystem, improving crop type and soil physical–chemical qualities may provide some management strategies for the functional diversity of soil bacteria and fungi.

## 5. Conclusions

By looking at factors of landscape complexity levels, crop type, soil fractions, and soil physical–chemical properties to study the composition and diversity of soil bacteria and fungi across the brassica cropping system, we showed that belowground ecology is very complex. This is probably the most extensive evidence of how complex the ecosystem is below the surface of the ground. The most abundant soil bacterial and fungal classes were Gammaproteobacteria and Sordriomycetes, respectively. In addition, the most abundant soil bacterial and fungal genera were Chuljaibacter and Penicillium, respectively. Out of the landscape composition, the proportion of brassica was the major factor for the bacterial–fungal beta diversity. The findings revealed that microbial richness is influenced in a near-uniform manner by the soil properties, soil fraction, crop type, and landscape composition. The importance of biotic and abiotic interplay in shaping the belowground biological diversity is emphasized. As a result, this study aids in gaining a better knowledge of the key soil bacterial and fungal groups in brassica cropping systems and their response to various landscape complexity levels, crop types, soil fractions, and soil physical–chemical parameters. This study will give us a valuable avenue for establishing better long-term management strategies for the brassica ecosystem and other vegetable crops.

## Figures and Tables

**Table 1:** The variance in assemblages of bacterial and fungal communities. ANOVA-like permutation test is used for RDA model to assess the significance of constraints. F represents test statistic (pseudo-F), which is the ratio of constrained and unconstrained total inertia (chi-squares or variance), each divided by their respective ranks.

Variables	Bacteria								Fungi							
	Genus		Family		Order		Class		Genus		Family		Order		Class	
	*F*	*p*-Value	*F*	*p*-Value	*F*	*p*-Value	*F*	*p*-Value	*F*	*p*-Value	*F*	*p*-Value	*F*	*p*-Value	*F*	*p*-Value
RDA Models	34.634	0.001	34.883	0.001	27.976	0.001	23.181	0.001	38.746	0.001	38.474	0.001	37.994	0.001	38.153	0.001
*Physical–chemical properties*																
Phosphorus (P)	19.214	0.001	19.7822	0.001	14.9689	0.001	18.5632	0.001	28.3599	0.001	20.9856	0.001	20.5855	0.001	16.2982	0.001
Zinc (Zn)	50.1576	0.001	56.7291	0.001	42.674	0.001	25.7486	0.001	76.3496	0.001	68.1285	0.001	69.0908	0.001	80.1055	0.001
Manganese (Mn)	128.0654	0.001	133.6238	0.001	97.6005	0.001	62.3831	0.001	86.8815	0.001	84.988	0.001	84.6954	0.001	98.778	0.001
Iron (Fe)	4.87	0.003	4.5214	0.006	4.6515	0.002	3.5794	0.007	17.8615	0.001	13.8971	0.001	14.9338	0.001	11.0018	0.001
Potassium (K)	32.2536	0.001	27.3877	0.001	26.5556	0.001	24.7061	0.001	60.6288	0.001	76.7898	0.001	80.2399	0.001	72.6909	0.001
Nitrogen (N)	4.2549	0.007	6.6598	0.001	6.5038	0.003	4.992	0.003	10.7669	0.001	11.7058	0.001	12.6321	0.001	17.9998	0.001
Calcium (Ca)	39.5991	0.001	34.0089	0.001	30.0193	0.001	27.4398	0.001	28.406	0.001	28.6641	0.001	30.827	0.001	26.0269	0.001
*Land use variables*																
Brassica	19.1152	0.001	16.7318	0.001	13.1291	0.001	5.5959	0.001	41.9085	0.001	42.9349	0.001	40.7998	0.001	44.5503	0.001
Non-brassica	17.4948	0.001	12.0336	0.001	9.4166	0.001	10.5305	0.001	20.4728	0.001	15.9562	0.001	19.4612	0.001	15.9813	0.001
Cultivated	68.6182	0.001	71.9228	0.001	55.3713	0.001	43.1516	0.001	39.5823	0.001	53.9929	0.001	51.8795	0.001	41.8935	0.001
Fallow	28.9311	0.001	26.7255	0.001	19.0214	0.001	21.5353	0.001	35.8917	0.001	18.0619	0.001	18.1037	0.001	13.3909	0.001
Forest	52.392	0.001	36.0979	0.001	30.7278	0.001	18.8807	0.001	21.662	0.001	21.7392	0.001	22.784	0.001	25.6496	0.001
Grassland	4.7679	0.003	5.8625	0.002	5.6152	0.001	3.5213	0.006	4.6772	0.001	8.8551	0.001	9.3071	0.001	9.7474	0.001
*Local variables*																
Host plant	11.7741	0.001	29.9131	0.001	21.128	0.001	29.5702	0.001	82.7351	0.001	79.338	0.001	65.4933	0.001	69.0251	0.001
Soil type	37.9961	0.001	41.2481	0.001	42.2607	0.001	47.5125	0.001	25.0033	0.001	31.0807	0.001	29.0829	0.001	29.1517	0.001

## Data Availability

Raw sequence is available publicly at NCBI database with accession numbers SAMN21466482-SAMN21466517 and SAMN21467089-SAMN21467124 for 16S and SAMN21467213-SAMN21467248 and SAMN21467258-SAMN21467293 for ITS. Datasets and R codes are available upon request.

## Supplementary Materials

The following supporting information can be downloaded at: https://www.mdpi.com/article/10.3390/microorganisms10061202/s1, Table S1. Mean and range (min-max)—of different vegetation types surrounding the focal crop field (%) within 100, 200, 300, 400 and 500 m radiuses of each sampling site; Table S2. Soil physiochemical properties in each sampling site; Table S3. Linear response results of alpha diversity indices against different host plant and soil types and their interaction; Table S4. ANOVA results of each axis in RDA model explaining the variance in assemblages of bacteria and fungi; Figure S1. Barplot shows different percentage proportion of different land uses at different sampling sites. The land uses include cultivated land, built-up, water, forest, grassland, fallow, brassica and non-brassica. The radiuses within 100, 200, 300, 400 and 500 around the focal patch of different sampling sites were studied; Figure S2. Barplot shows the relative occurrences of soil physiochemical elements in different fields. Elements consist of calcium, iron, potassium, manganese, nitrogen, phosphorus, zinc, magnesium and copper; Figure S3. Relative abundance (%) of different soil bacterial and fungal communities in bulk and rhizospheric soils collected from Chinese cabbage and flower cabbage fields; Figure S4. Redundancy analysis (RDA), illustrating the effects of landscape factors, soil physical-chemical properties, soil fraction and crop type.
