# Supplementary material for: Landscape Composition and Soil Physical–Chemical Properties Drive the Assemblages of Bacteria and Fungi in Conventional Vegetable Fields
Source: Microorganisms. 2022 Jun 12;10(6):1202. doi: 10.3390/microorganisms10061202 (PMC9229475; doi:10.3390/microorganisms10061202)
Supplement: Supplementary file 1 [file microorganisms-10-01202-s001.zip › microorganisms-1757373-supplementary.pdf]

Supplementary Material

Landscape Composition and Soil Physical-chemical Properties Drive the Assemblages of Bacteria and fungi in Conventional Vegetable Fields

Uttam Kumar [1,2,3,†], Hafiz Sohaib Ahmed Saqib [1,2,3,4,†,*], Waqar Islam [5,6], Parmar Prashant [1,2,3], Nidhibahen Patel [1,2,3], Wei Chen [1,2,3], Feiying Yang [1,2,3,7], Minsheng You [1,2,3], Weyi He [1,2,3,*]

[1] State Key Laboratory of Ecological Pest Control for Fujian and Taiwan Crops, Institute of Applied Ecology, Fujian Agriculture and Forestry University, Fuzhou 350002, China

[2] Joint International Research Laboratory of Ecological Pest Control, Ministry of Education, Fuzhou 350002, China

[3] Ministerial and Provincial Joint Innovation Centre for Safety Production of Cross-Strait Crops, Fujian Agriculture and Forestry University, Fuzhou 350002, China

[4] Guangdong Provincial Key Laboratory of Marine Biology, College of Science, Shantou University, Shantou 515063, China

[5] Institute of Geography, Fujian Normal University, Fuzhou, 350007, China

[6] University of Chinese Academy of Sciences, Beijing, 100049, China

[7] Institute of Horticultural Sciences, Jiangxi Academy of Agricultural Sciences, Nanchang 330200, China

[†] These authors have contributed equally to this work and share first authorship.

[*] **Correspondence:** wy.he@fafu.edu.cn (W.H.); saqib@stu.edu.cn (H.S.A.S)

Table S1 Mean and range (min-max)—of different vegetation types surrounding the focal crop field (%) within 100, 200, 300, 400 and 500 m radiuses of each sampling site.

Site name	Radius	Land uses							
		Brassica	Built-up	Fallow	Cultivated	Forest	Grassland	Non-brassica	Water
Dahuxiang-1	100m	31.95(4.03-36.61)	5.67(2.53-24.51)	0.01(0-0.1)	14.72(5.75-16.21)	34.48(29.77-62.75)	12.84(0.62-14.88)	0(0-0)	0.32(0-2.25)
Dahuxiang-1	200m	11.21(4.69-12.3)	6.34(4.77-15.74)	0.01(0-0.06)	23.29(14.42-24.77)	53.4(52.56-58.44)	5.38(4.04-5.6)	0(0-0)	0.37(0-2.61)
Dahuxiang-1	300m	5.76(2.93-6.23)	6.74(4.72-18.83)	0.1(0-0.73)	24.14(16.22-25.46)	56.56(48.97-57.83)	4.14(2.99-11.03)	0(0-0)	2.55(1.29-2.76)
Dahuxiang-1	400m	3.85(2.54-4.06)	6.67(5.11-16.04)	0.14(0-0.97)	24.27(22.19-24.62)	58.78(44.39-61.18)	3.64(2.23-12.07)	0.02(0-0.14)	2.63(1.65-2.8)
Dahuxiang-1	500m	3.18(2.49-3.3)	6.76(5.49-14.38)	0.35(0.27-0.85)	22.82(21.9-28.29)	61.18(39.41-64.81)	3.67(2.14-12.84)	0.04(0-0.25)	2.01(1.48-2.09)
Dahuxiang-2	100m	4.03(4.03-4.03)	24.51(24.51-24.51)	0.1(0.1-0.1)	5.75(5.75-5.75)	62.75(62.75-62.75)	0.62(0.62-0.62)	0(0-0)	2.25(2.25-2.25)
Dahuxiang-2	200m	4.69(4.69-4.69)	15.74(15.74-15.74)	0.06(0.06-0.06)	14.42(14.42-14.42)	58.44(58.44-58.44)	4.04(4.04-4.04)	0(0-0)	2.61(2.61-2.61)
Dahuxiang-2	300m	2.93(2.93-2.93)	18.83(18.83-18.83)	0.73(0.73-0.73)	16.22(16.22-16.22)	48.97(48.97-48.97)	11.03(11.03-11.03)	0(0-0)	1.29(1.29-1.29)
Dahuxiang-2	400m	2.54(2.54-2.54)	16.04(16.04-16.04)	0.97(0.97-0.97)	22.19(22.19-22.19)	44.39(44.39-44.39)	12.07(12.07-12.07)	0.14(0.14-0.14)	1.65(1.65-1.65)
Dahuxiang-2	500m	2.49(2.49-2.49)	14.38(14.38-14.38)	0.85(0.85-0.85)	28.29(28.29-28.29)	39.41(39.41-39.41)	12.84(12.84-12.84)	0.25(0.25-0.25)	1.48(1.48-1.48)
Dahuxiang-3	100m	15.96(15.96-15.96)	33.65(33.65-33.65)	0(0-0)	46.69(46.69-46.69)	0.82(0.82-0.82)	1.82(1.82-1.82)	0(0-0)	1.06(1.06-1.06)
Dahuxiang-3	200m	2.25(2.25-2.25)	4.14(4.14-4.14)	0(0-0)	15.85(15.85-15.85)	75.51(75.51-75.51)	1.47(1.47-1.47)	0(0-0)	0.78(0.78-0.78)
Dahuxiang-3	300m	3.31(3.31-3.31)	3.41(3.41-3.41)	0(0-0)	22(22-22)	65.77(65.77-65.77)	4.86(4.86-4.86)	0(0-0)	0.65(0.65-0.65)
Dahuxiang-3	400m	3(3-3)	3.82(3.82-3.82)	0(0-0)	26.7(26.7-26.7)	59.85(59.85-59.85)	6.08(6.08-6.08)	0(0-0)	0.56(0.56-0.56)
Dahuxiang-3	500m	3.45(3.45-3.45)	3.75(3.75-3.75)	0(0-0)	28.54(28.54-28.54)	56.01(56.01-56.01)	7.34(7.34-7.34)	0.08(0.08-0.08)	0.82(0.82-0.82)
Guangzhuang	100m	14.79(14.79-14.79)	8.38(8.38-8.38)	0(0-0)	2.93(2.93-2.93)	51.74(51.74-51.74)	4.78(4.78-4.78)	4.57(4.57-4.57)	12.8(12.8-12.8)
Guangzhuang	200m	22.03(22.03-22.03)	18.03(18.03-18.03)	0(0-0)	5.38(5.38-5.38)	33.4(33.4-33.4)	3.74(3.74-3.74)	9.16(9.16-9.16)	8.26(8.26-8.26)
Guangzhuang	300m	23.17(23.17-23.17)	25.79(25.79-25.79)	0(0-0)	5.08(5.08-5.08)	23.91(23.91-23.91)	4.25(4.25-4.25)	12.33(12.33-12.33)	5.46(5.46-5.46)
Guangzhuang	400m	19.56(19.56-19.56)	28.1(28.1-28.1)	2.64(2.64-2.64)	3.96(3.96-3.96)	26.45(26.45-26.45)	5.88(5.88-5.88)	9.5(9.5-9.5)	3.91(3.91-3.91)
Guangzhuang	500m	20.85(20.85-20.85)	26.67(26.67-26.67)	2.7(2.7-2.7)	3.45(3.45-3.45)	24.8(24.8-24.8)	9.23(9.23-9.23)	8.7(8.7-8.7)	3.59(3.59-3.59)
Nantong-1	100m	6.17(6.17-6.17)	48.9(48.9-48.9)	0(0-0)	4.92(4.92-4.92)	15.6(15.6-15.6)	0(0-0)	11.87(11.87-11.87)	12.54(12.54-12.54)
Nantong-1	200m	3.85(3.85-3.85)	40.3(40.3-40.3)	0(0-0)	6.47(6.47-6.47)	23.36(23.36-23.36)	0.64(0.64-0.64)	12.71(12.71-12.71)	12.68(12.68-12.68)
Nantong-1	300m	3.47(3.47-3.47)	41.11(41.11-41.11)	1.4(1.4-1.4)	5.72(5.72-5.72)	19.91(19.91-19.91)	1.18(1.18-1.18)	13.82(13.82-13.82)	13.39(13.39-13.39)
Nantong-1	400m	3.05(3.05-3.05)	38.25(38.25-38.25)	1.43(1.43-1.43)	6.29(6.29-6.29)	17.19(17.19-17.19)	5.52(5.52-5.52)	16.81(16.81-16.81)	11.47(11.47-11.47)
Nantong-1	500m	3.02(3.02-3.02)	35.61(35.61-35.61)	1.55(1.55-1.55)	7.81(7.81-7.81)	16.43(16.43-16.43)	5.92(5.92-5.92)	18.48(18.48-18.48)	11.18(11.18-11.18)
Nantong-2	100m	17.43(17.43-17.43)	76.58(76.58-76.58)	0(0-0)	1.61(1.61-1.61)	0(0-0)	3.11(3.11-3.11)	1.27(1.27-1.27)	0(0-0)
Nantong-2	200m	28.11(28.11-28.11)	44.14(44.14-44.14)	0(0-0)	1.67(1.67-1.67)	6.36(6.36-6.36)	1.81(1.81-1.81)	3.5(3.5-3.5)	14.41(14.41-14.41)
Nantong-2	300m	28.29(28.29-28.29)	40.96(40.96-40.96)	0(0-0)	2.1(2.1-2.1)	4.88(4.88-4.88)	1.13(1.13-1.13)	8.29(8.29-8.29)	14.35(14.35-14.35)
Nantong-2	400m	32.19(32.19-32.19)	33.42(33.42-33.42)	0(0-0)	3.02(3.02-3.02)	6.62(6.62-6.62)	1.25(1.25-1.25)	12.07(12.07-12.07)	11.42(11.42-11.42)
Nantong-2	500m	36.65(36.65-36.65)	28.8(28.8-28.8)	0(0-0)	4.15(4.15-4.15)	6.23(6.23-6.23)	1.34(1.34-1.34)	12.77(12.77-12.77)	10.06(10.06-10.06)

Nantong-3	100m	1.96(1.96-1.96)	5.96(5.96-5.96)	0(0-0)	0(0-0)	87.57(87.57-87.57)	1.18(1.18-1.18)	3.02(3.02-3.02)	0.31(0.31-0.31)
Nantong-3	200m	3(3-3)	6(6-6)	0(0-0)	0(0-0)	83.1(83.1-83.1)	2.93(2.93-2.93)	4.48(4.48-4.48)	0.49(0.49-0.49)
Nantong-3	300m	5.18(5.18-5.18)	5.67(5.67-5.67)	0(0-0)	0.58(0.58-0.58)	78.65(78.65-78.65)	3.86(3.86-3.86)	4.84(4.84-4.84)	1.23(1.23-1.23)
Nantong-3	400m	5.48(5.48-5.48)	5.94(5.94-5.94)	0(0-0)	0.51(0.51-0.51)	79.4(79.4-79.4)	3.4(3.4-3.4)	4.21(4.21-4.21)	1.07(1.07-1.07)
Nantong-3	500m	6.57(6.57-6.57)	5.88(5.88-5.88)	0(0-0)	0.5(0.5-0.5)	78.28(78.28-78.28)	3.58(3.58-3.58)	4.14(4.14-4.14)	1.05(1.05-1.05)
Nantong-4	100m	19.07(19.07-19.07)	66(66-66)	0(0-0)	1.48(1.48-1.48)	0(0-0)	2.69(2.69-2.69)	0(0-0)	10.75(10.75-10.75)
Nantong-4	200m	31.23(31.23-31.23)	46.26(46.26-46.26)	0(0-0)	1.36(1.36-1.36)	0(0-0)	7.4(7.4-7.4)	5.88(5.88-5.88)	7.88(7.88-7.88)
Nantong-4	300m	27.2(27.2-27.2)	28.87(28.87-28.87)	0(0-0)	1.35(1.35-1.35)	16.37(16.37-16.37)	8.81(8.81-8.81)	7.61(7.61-7.61)	9.79(9.79-9.79)
Nantong-4	400m	32.76(32.76-32.76)	22.34(22.34-22.34)	0(0-0)	1.46(1.46-1.46)	12.66(12.66-12.66)	13.92(13.92-13.92)	7.73(7.73-7.73)	9.12(9.12-9.12)
Nantong-4	500m	31.36(31.36-31.36)	20.66(20.66-20.66)	0(0-0)	4.41(4.41-4.41)	11.31(11.31-11.31)	14.62(14.62-14.62)	7.27(7.27-7.27)	10.37(10.37-10.37)
Nantong-5	100m	16.88(16.88-16.88)	20.75(20.75-20.75)	0(0-0)	0(0-0)	43.96(43.96-43.96)	3.2(3.2-3.2)	0(0-0)	15.22(15.22-15.22)
Nantong-5	200m	19.34(19.34-19.34)	25.56(25.56-25.56)	0(0-0)	0(0-0)	28.15(28.15-28.15)	3.06(3.06-3.06)	2.63(2.63-2.63)	21.27(21.27-21.27)
Nantong-5	300m	19.61(19.61-19.61)	16.64(16.64-16.64)	0(0-0)	0(0-0)	25.6(25.6-25.6)	7.58(7.58-7.58)	4.85(4.85-4.85)	25.73(25.73-25.73)
Nantong-5	400m	22.16(22.16-22.16)	13.5(13.5-13.5)	0(0-0)	0(0-0)	27.03(27.03-27.03)	9.39(9.39-9.39)	3.57(3.57-3.57)	24.35(24.35-24.35)
Nantong-5	500m	23.91(23.91-23.91)	12.52(12.52-12.52)	0(0-0)	0(0-0)	27.15(27.15-27.15)	9.8(9.8-9.8)	3.37(3.37-3.37)	23.26(23.26-23.26)
Xiaomifeng	100m	1.6(1.6-1.6)	4.17(4.17-4.17)	0(0-0)	0(0-0)	88.13(88.13-88.13)	5.2(5.2-5.2)	0.7(0.7-0.7)	0.2(0.2-0.2)
Xiaomifeng	200m	1.91(1.91-1.91)	2.21(2.21-2.21)	23.02(23.02-23.02)	0(0-0)	64.55(64.55-64.55)	5.09(5.09-5.09)	2.91(2.91-2.91)	0.31(0.31-0.31)
Xiaomifeng	300m	1.71(1.71-1.71)	2.72(2.72-2.72)	20.62(20.62-20.62)	0(0-0)	63.62(63.62-63.62)	5.87(5.87-5.87)	5.19(5.19-5.19)	0.27(0.27-0.27)
Xiaomifeng	400m	1.56(1.56-1.56)	2.89(2.89-2.89)	18.84(18.84-18.84)	0(0-0)	64.39(64.39-64.39)	5.52(5.52-5.52)	6.38(6.38-6.38)	0.42(0.42-0.42)
Xiaomifeng	500m	1.51(1.51-1.51)	3.04(3.04-3.04)	18.22(18.22-18.22)	0(0-0)	63.6(63.6-63.6)	6.4(6.4-6.4)	6.83(6.83-6.83)	0.4(0.4-0.4)
Xinan	100m	40.03(40.03-40.03)	31.31(31.31-31.31)	0(0-0)	26.46(26.46-26.46)	0(0-0)	0.08(0.08-0.08)	2.11(2.11-2.11)	0(0-0)
Xinan	200m	16.24(16.24-16.24)	31.43(31.43-31.43)	2.72(2.72-2.72)	16.1(16.1-16.1)	31.25(31.25-31.25)	1.53(1.53-1.53)	0.39(0.39-0.39)	0.35(0.35-0.35)
Xinan	300m	10.4(10.4-10.4)	25.87(25.87-25.87)	4.06(4.06-4.06)	12.45(12.45-12.45)	32.17(32.17-32.17)	3.92(3.92-3.92)	0.5(0.5-0.5)	10.63(10.63-10.63)
Xinan	400m	8.58(8.58-8.58)	22.87(22.87-22.87)	4.15(4.15-4.15)	12.25(12.25-12.25)	34.41(34.41-34.41)	5.88(5.88-5.88)	0.35(0.35-0.35)	11.51(11.51-11.51)
Xinan	500m	8.19(8.19-8.19)	22.41(22.41-22.41)	5.69(5.69-5.69)	12.33(12.33-12.33)	34.08(34.08-34.08)	6.56(6.56-6.56)	0.32(0.32-0.32)	10.42(10.42-10.42)
Xiqian	100m	9.64(9.64-9.64)	50.53(50.53-50.53)	0(0-0)	25.89(25.89-25.89)	0(0-0)	13.48(13.48-13.48)	0(0-0)	0.46(0.46-0.46)
Xiqian	200m	8.97(8.97-8.97)	20.57(20.57-20.57)	0(0-0)	19.86(19.86-19.86)	0.07(0.07-0.07)	33.41(33.41-33.41)	0(0-0)	17.13(17.13-17.13)
Xiqian	300m	6.64(6.64-6.64)	17.59(17.59-17.59)	0(0-0)	18.65(18.65-18.65)	24.67(24.67-24.67)	23.26(23.26-23.26)	0.08(0.08-0.08)	9.11(9.11-9.11)
Xiqian	400m	8.51(8.51-8.51)	16.53(16.53-16.53)	0(0-0)	17.54(17.54-17.54)	29.85(29.85-29.85)	19.06(19.06-19.06)	0.21(0.21-0.21)	8.31(8.31-8.31)
Xiqian	500m	9.28(9.28-9.28)	18.24(18.24-18.24)	0(0-0)	17.71(17.71-17.71)	28.8(28.8-28.8)	18.04(18.04-18.04)	0.88(0.88-0.88)	7.06(7.06-7.06)

Non-cruciferous vegetables include eggplant, pepper, tomatoes, bean, and corn.

Forest include coniferous, bamboo and broad-leaved trees.

Grassland include the strip and patch grassland in cultivated area and natural grass between forest and cultivated land.

Urban include residential district, road and artificial greenhouse.

Water include river, pond and reservoir.

Table S2 Soil physiochemical properties in each sampling site.

Region	Calcium (Ca)	Copper (Cu)	Iron (Fe)	Potassium (K)	Magnesium (Mg)	Manganese (Mn)	Nitrogen (N)	Phosphorus (P)	Zinc (Zn)
Dahuxiang-1	605.19±73.57	8.46±0.54	4645±538	4974.77±250.37	291.94±14.17	112.86±4.88	0.4±0.01	11331.97±102.92	32.71±2.35
Dahuxiang-2	701.16±45.56	10.95±0.41	2234±85.94	5561.48±20.23	338.88±7.16	143.5±3.88	0.36±0.01	11275.6±140.28	37.7±0.66
Dahuxiang-3	176.6±5.25	4.17±0.24	824.17±58.85	4334.11±54.94	113.5±2.44	90.21±3.89	0.33±0.02	7647.59±107.79	19.63±0.62
Guangzhuang	221.65±5.59	3.67±0.23	2037.5±121.95	6458.41±83.07	172.8±3.77	122.92±7.63	0.43±0.01	6432.73±68	34.71±2.13
Nantong-1	273.2±7.2	5.08±0.32	5569.17±279.05	7335.11±41.53	321.9±3.85	282.29±26.28	0.44±0.01	9791.16±94.84	43.75±0.83
Nantong-2	224.15±15.08	14.21±1.22	5299.17±356.87	6846.18±125.43	311.75±7.48	347.29±31.48	21.93±8.98	7085.34±447.48	43.71±1.73
Nantong-3	200.15±7.45	3.42±0.33	1473.33±127.38	5784.03±28.69	172.6±5.9	156.25±9.12	0.37±0.01	8719.38±463.61	28.5±1.05
Nantong-4	319.7±6.92	7.17±0.81	5486.67±174.65	7469.98±59.07	410.7±1.71	505.63±20	0.42±0.01	2652.61±51.15	41.25±1.62
Nantong-5	251.7±19.28	15.08±1.24	7349.17±725.35	7082.21±126.2	343.45±2.55	285±10.81	0.31±0.03	7853.41±514.55	57.42±0.71
Xiaomifeng	520.9±23.7	38.96±0.71	5451.67±259.57	4114.93±51.73	286.4±5.35	543.75±39.81	0.34±0.02	1198859.94±495736.52	67.46±1.22
Xinan	394.15±18.04	4.54±0.14	2355.83±186.07	4249.81±29.54	145.4±1.91	142.71±13.08	0.37±0.02	8217.37±395.43	18.58±0.41
Xiqian	520.95±6.15	20.29±0.41	7610±609.48	3423.69±20.77	321.15±6.25	266.67±14.09	0.42±0.01	3247.49±96.87	59.08±1.63

Table S3 Linear response results of alpha diversity indices against different host plant and soil types and their interaction.

Kingdom	Factors	Chao1		ACE		Shannon	
		F-value	*P*-value	F-value	*P*-value	F-value	*P*-value
Bacteria	Host plant (HP)	6.340	0.013	8.813	0.003	5.834	0.017
	Soil type (ST)	7.911	0.006	13.724	0.000	0.084	0.772
	HP*ST	29.567	0.000	18.650	0.000	21.741	0.000
Fungi	Host plant (HP)	1.255	0.264	3.009	0.085	12.444	0.001
	Soil type (ST)	33.805	0.000	32.342	0.000	16.428	0.000
	HP*ST	8.947	0.003	12.515	0.001	2.559	0.112

Table S4 ANOVA results of each axis in RDA model explaining the variance in assemblages of bacteria and fungi.

RDA axis	Bacteria		Fungi	
	F-value	*P*-value	F-value	*P*-value
RDA1	142.717	0.001	364.828	0.001
RDA2	107.015	0.001	116.32	0.001
RDA3	41.6626	0.001	44.709	0.001
RDA4	18.6081	0.001	17.1641	0.001
RDA5	14.1936	0.001	12.4698	0.001
RDA6	8.9764	0.003	5.7999	0.057
RDA7	7.0576	0.012	5.3179	0.075
RDA8	3.5728	0.44	2.2123	0.928
RDA9	1.604	0.986	1.5693	0.993
RDA10	1.0745	0.999	0.8894	0.999
RDA11	0.5652	1	0.7303	1
RDA12	0.3339	1	0.2118	1
RDA13	0.2088	1	0.0548	1
RDA14	0.094	1	0.0145	1
RDA15	0.0267	1	0.0002	1

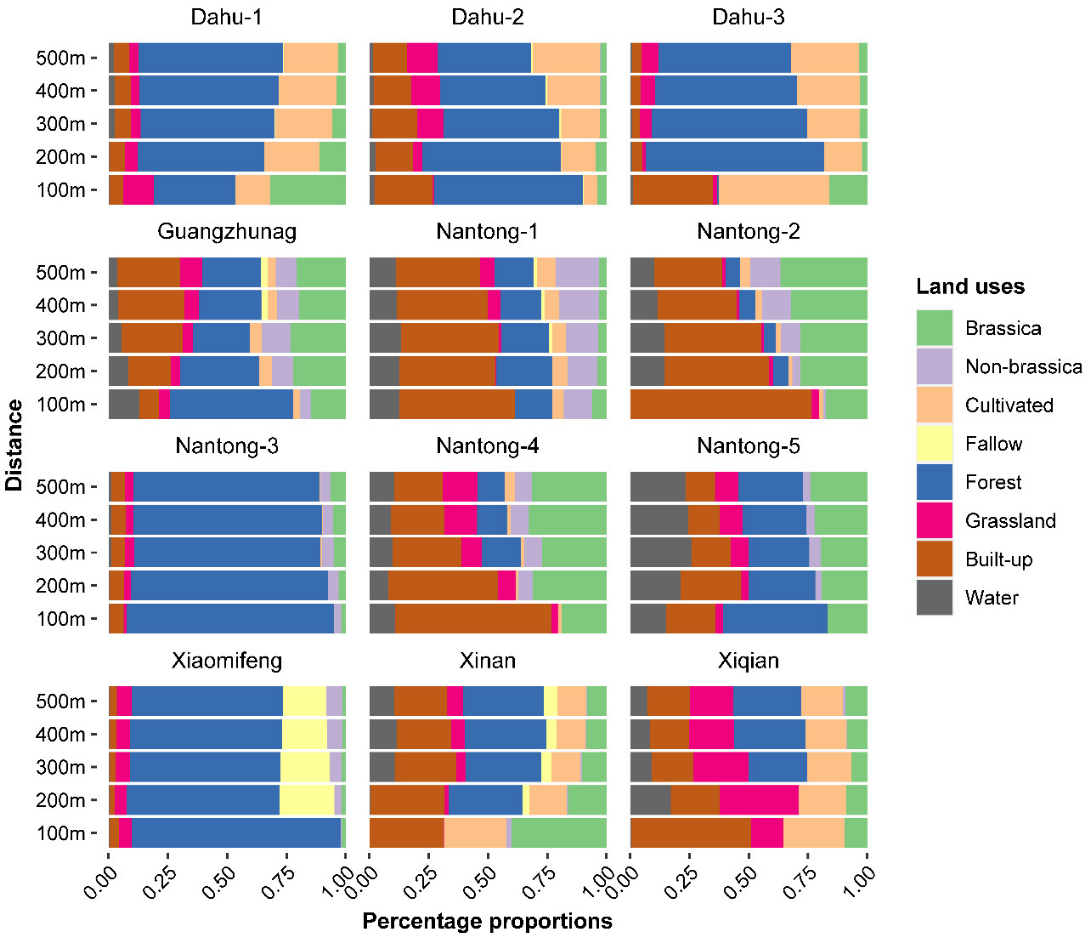

Figure S1 Barplot shows different percentage proportion of different land uses at different sampling sites. The land uses include cultivated land, built-up, water, forest, grassland, fallow, brassica and non-brassica. The radiuses within 100, 200, 300, 400 and 500 around the focal patch of different sampling sites were studied.

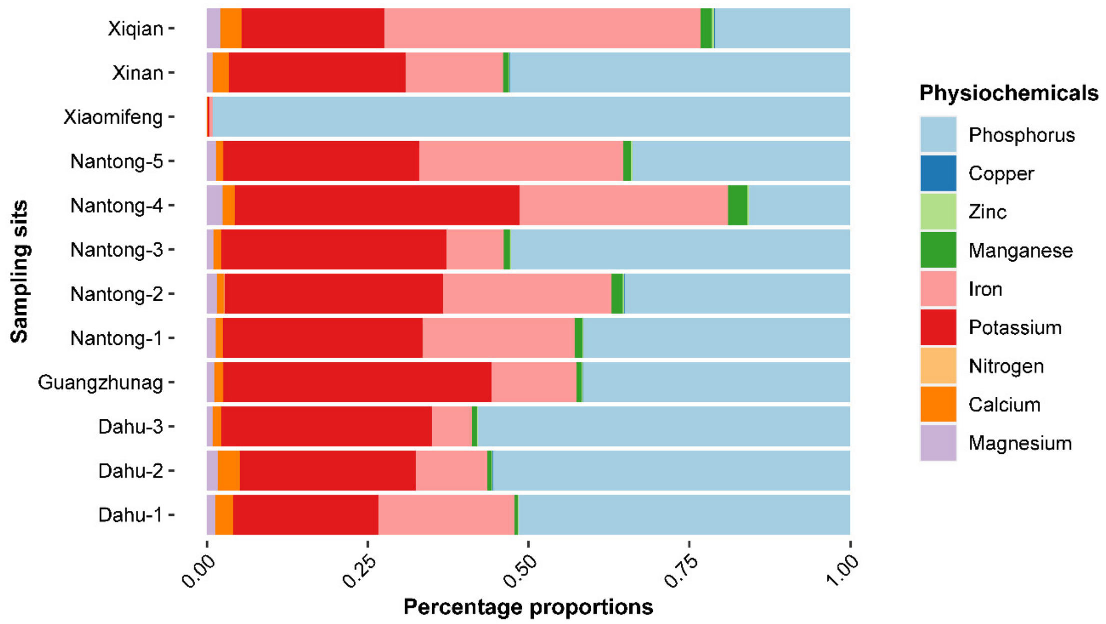

Figure S2 Barplot shows the relative occurrences of soil physiochemical elements in different fields. Elements consist of calcium, iron, potassium, manganese, nitrogen,

phosphorus, zinc, magnesium and copper.

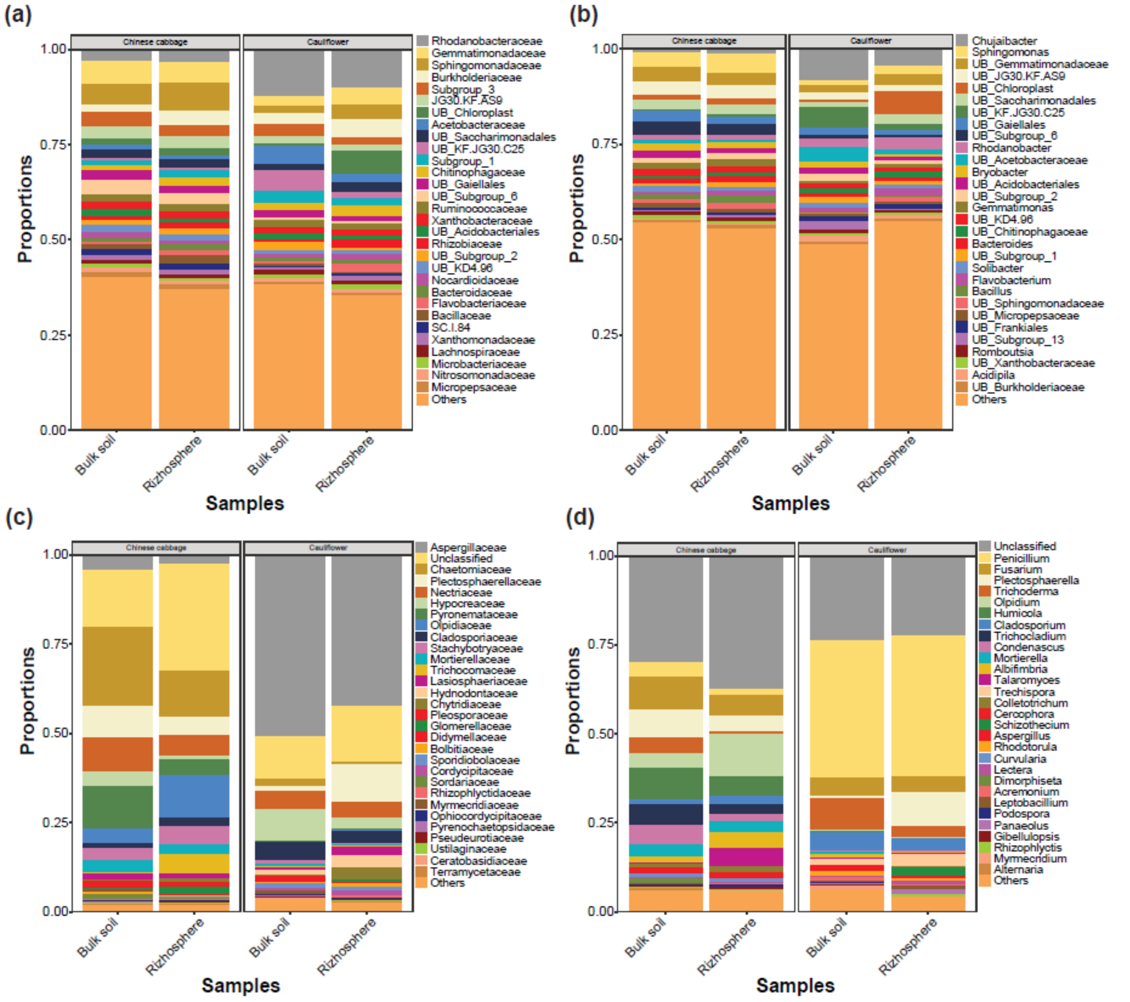

Figure S3. Relative abundance (%) of different soil bacterial and fungal communities in bulk and rhizospheric soils collected from Chinese cabbage and flower cabbage fields. (**a**) Relative abundance of bacteria at family level; (**b**) Relative abundance of fungi at family level; (**c**) Relative abundance of bacteria at genus level; (**d**) Relative abundance of fungi at genus level.

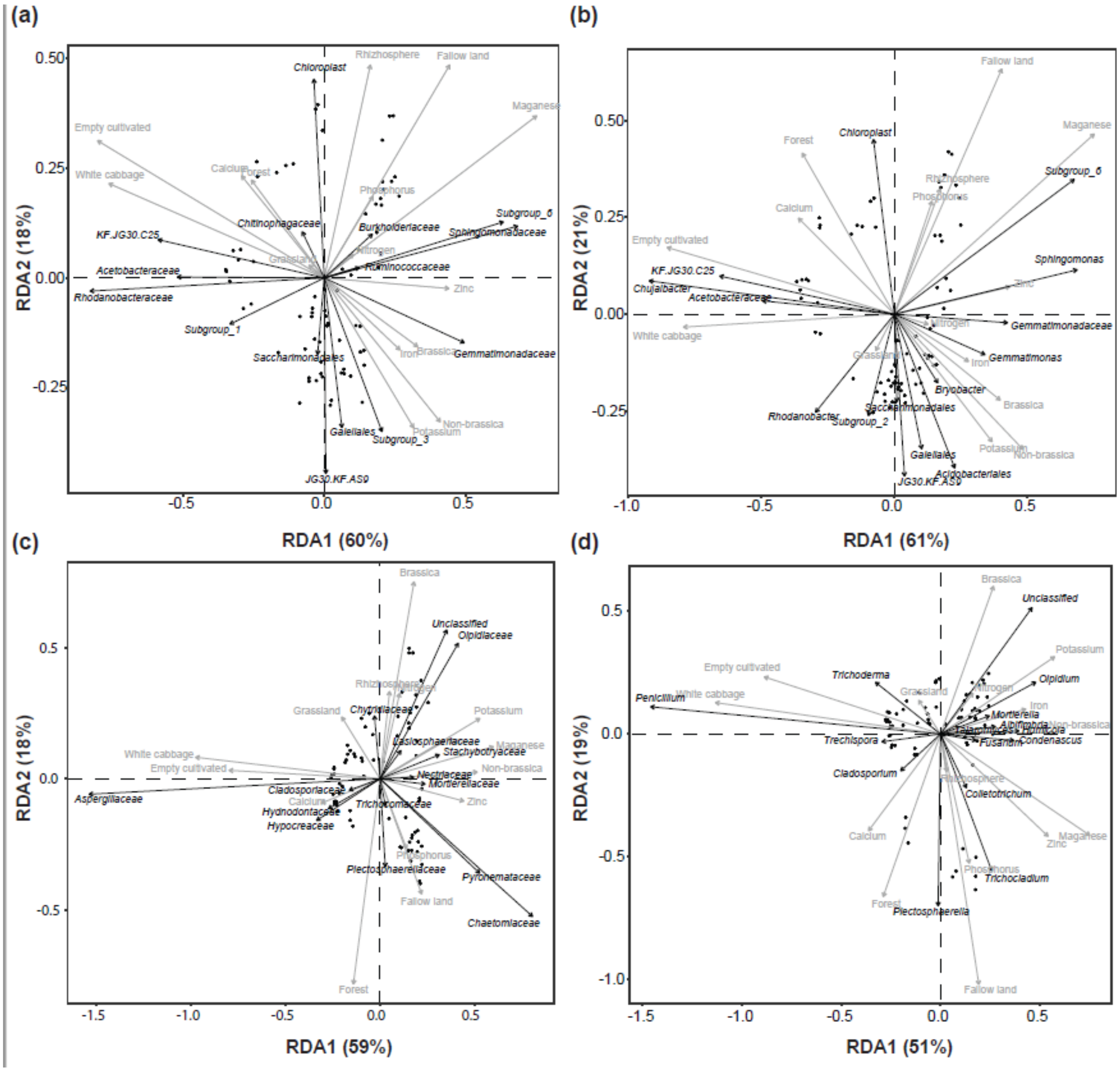

Figure S4. Redundancy analysis (RDA), illustrating the effects of landscape factors, soil physical-chemical properties, soil fraction and crop type (arrows) on (**a**) soil bacterial families, (**b**) soil bacterial genera (**c**) soil fungal families (**d**) soil fungal genera. The length and orientation of the arrows show the amount of variance that the explanatory and response variables can explain. The correlations between soil bacterial and fungal classes and explanatory variables are represented by the perpendicular distance between them (<90° = positive correlation, > 90° = negative correlation). The stronger association is represented by smaller perpendicular distance.
